# Exploring Heat Stress Relief Measures among the Australian Labour Force

**DOI:** 10.3390/ijerph15030401

**Published:** 2018-02-26

**Authors:** Kerstin K. Zander, Supriya Mathew, Stephen T. Garnett

**Affiliations:** 1 Northern Institute, Charles Darwin University, Darwin, NT 0909, Australia; Supriya.Mathew@cdu.edu.au; 2 Research Institute for the Environment and Livelihoods, Charles Darwin University, Darwin, NT 0909, Australia; stephen.garnett@cdu.edu.au

**Keywords:** acclimatisation, heat stress, health and safety, public health, online survey

## Abstract

Australia experiences frequent heat waves and generally high average temperatures throughout the continent with substantial impacts on human health and the economy. People adapt to heat by adopting various relief measures in their daily lives including changing their behaviour. Many labour intensive outdoor industries implement standards for heat stress management for their workforce. However, little is known about how people cope with heat at their workplaces apart from studies targeting some specific industries where labourers are exposed to extreme heat. Here, we analysed responses from 1719 people in the Australian labour force to self-reported heat stress and associated coping mechanisms. Three quarters of respondents experienced heat stress at their workplace with fatigue and headache being the two most frequently stated symptoms. Almost all of those who were affected by heat would hydrate (88%), 67% would cool, and 44% would rest as a strategy for coping with heat. About 10% intended to change their jobs because of heat stress in the workplace. We found differences in heat relief measures across gender, education, health, level of physical intensity of job, and time spent working outside. People working in jobs that were not very demanding physically were more likely to choose cooling down as a relief measure, while those in labour intensive jobs and jobs that required considerable time outside were more likely to rest. This has potential consequences for their productivity and work schedules. Heat affects work in Australia in many types of industry with impact dependent on workforce acclimatisation, yet public awareness and work relief plans are often limited to outdoor and labour intensive industries. Industries and various levels of government in all sectors need to implement standards for heat management specific to climate zones to help people cope better with high temperatures as well as plan strategies in anticipation of projected temperature increases.

## 1. Introduction

Occupational health issues from heat have long been a concern for occupations involving intense physical labour and or exposure to high temperatures. Workers in heat-affected sectors and occupations have been identified as being vulnerable to heat stress [1] and have been researched increasingly in the last decade [2,3,4,5,6,7,8]. The health effects of heat in Australia are diverse, ranging from aggravated cardio-respiratory-renal issues to preterm deliveries [9,10,11,12]. People’s mental health and cognitive abilities can also suffer from heat [13,14] and heat can distort time perception [15] and decision quality [16,17].

Because heat also reduces worker productivity [18,19] and leads to higher work place accident rates [20,21,22], some sectors, such as mining, military and construction, have heat stress risk assessment, management and prevention plans in place, administered through the umbrella of “Health and Safety”. These guidelines often provide information about the length of time that workers can physically and mentally tolerate heat exposure [23]. Most of these also apply a precautionary principle and assume conservative thresholds at which exposure to heat is considered safe and manageable. However, in many occupations, there are no heat safety guidelines [23] although some countries issue guidelines for workers that are not sector-specific (e.g., in the U.S. [24]). Some Australian jurisdictions issue guidelines for the general public, not just the labour force, to prepare for and deal with heat waves (e.g., heat health plan for Victoria [25]). We are not aware of any literature regarding heat relief management of the general labour force which is not sector or occupation specific or of specific low intensity occupations (e.g., office jobs). 

This paper aims to explore the extent to which heat affects a broad sample of the Australian workforce, not just those in physically demanding or heat-exposed industries. We consider the most common symptoms that can affect people when exposed to extreme heat even if they are not clinically heat-stressed and also explore how heat-affected people adapt. In more detail, we explore how heat stress mediates heat relief and prevention measures across different industry sectors, workplace-related characteristics and other personal factors that influence these measures. Australia is an appropriate case study country for this research because it is one of the hottest and driest continents with the heat waves predicted to become more frequent and longer lasting [26] and annual average temperatures increasing [27]. Recent research from Australia has dealt with workers’ health and safety at times of heat waves [6,22], but not at times of high temperatures when not defined as a heat wave, i.e., sporadic very hot days. In addition, most of this existing research investigates secondary data such as workers compensation claims or hospital admissions. As this is not survey based, it can only link heat stress data to parameters such as age, gender and work sector and has no information on perceptions or behaviour.

## 2. Materials and Methods

### 2.1. Data Collection

A survey questionnaire was prepared to collect data from the Australian workforce on various demographic variables, working environments, overall health, experiences of heat stress and their severity as well as various measures adopted by the Australian workforce to relieve the effects of heat. We focused on three immediate relief measures that are currently used in occupational settings [23]: hydrating (drinking), cooling (without resting, i.e., continue working in cooler environment) and resting (stopping work). We added a fourth response, changing jobs, as the most extreme form of mitigating heat at work, and a long-term strategy rather than immediate relief measure. The questionnaire was pre-tested with ten employees from Charles Darwin University from various disciplines to check the clarity, wording and time to complete the questionnaire.

The main survey was delivered through a commissioned online survey in two stages, Wave 1 in May and Wave 2 in October 2014. The sample was drawn from a research company’s online panel (MyOpinions PermissionCorp., Chatswood, Australia) which includes about 300,000 verified respondents. This panel is continuously being developed and maintained, strictly “research only”, and with recruitment coming from a wide range of offline and online media sources. The research company sent out email invitations to a random sub-sample of their panel of people over 18 years. The invitations contained the link to our survey, information about the time needed to complete the questions and the size of the incentive (respondents were paid the equivalent of US$2 on completion). No information about the topic of the survey was revealed in the invitation to avoid decisions to participate based on research topic. Those people who decided to participate then followed the link to the first page of our survey which contained information about the research scope and aims, and the research team. This first page also stated that the survey was voluntary, that the data obtained were non-identifiable and only used for research purpose. It also contained contact information should respondents have wanted to make comments or complaints. Respondents gave consent by proceeding from the first page and starting with the first question. We obtained approval to collect data from the Charles Darwin University Human Research Ethics Committee (H13119).

The research company invited 9405 people from their panel (4912 for Wave 1 and 4493 for Wave 2), and 22.2% of invited respondents started the survey. No information is available on the non-respondents but, as they had no knowledge of the content of the survey before they decided not to respond, non-response cannot be associated with any variable of interest. Of those starting the survey, close to 10% dropped out before completing the survey, leaving 1877 completed responses, 799 from Wave 1 and 1078 from Wave 2 (see Table S1). The final response rate of 18.3% is acceptable for online surveys, whose response rates are lower than for mail-out paper surveys [28].

A few of these 1877 responses (158) could not be used for analysis because the time of completion was less than 5 min or answers were inconsistent, indicating that respondents did not look at the questions seriously or did not understand them. The final dataset contained 1719 responses.

### 2.2. Analysis

Statistical analysis was conducted in R (R Core Team, Vienna, Austria) [29]. Logit models were used with each of the heat relief measures as dependent variables. As explanatory variables we included the level of heat stress, variables that describing the workplaces (work-related), variables describing people’s background (personal control variables), perceptions and attitudes and the location at which people lived (Table S2). Four personal control variables were included in the models: gender, age, level of education, and health status. These were selected because previous studies have shown that they have a direct impact on human heat stress [30,31] and might therefore also affect how people cope with heat. Before running the models, we tested if the independent variables to be included in the models were highly correlated, with a threshold of 0.70. We found no high correlations (Table S4). We then estimated the saturated models with all variables included, as well as interaction effects between gender and physical exertion and age and health (see Tables S5–S8), and then used the stepwise function (backwards and forward) to find the final model (based on BIC). We present the odds ratios (OR) as results with 95% confidence intervals (CI).

Bivariate relationships were analysed using ANOVA with subsequent pairwise comparisons of means (Tukey test) and non-parametric Kruskal–Wallis tests with pairwise comparisons of means after Nemenyi. ANOVA was used determine if there were statistically significant differences between groups of people with different levels of heat stress (independent variable: heat stress category) on continuous dependent variables. Kruskal–Wallis tests were used instead when the dependent variables were categorical, i.e., when the assumptions required for ANOVA were not met. The assumption of normality was tested using the Shapiro–Wilk, and the assumption of homogeneity of variances using the Levene’s test.

## 3. Results

### 3.1. Sample Characteristics

Of all 1719 respondents, 48% were female and the average age 40.5 (SD: 12.2) with a median of 40 years, slightly higher than the national median age of 37 years [32]. The average annual personal income before tax was AUD 61,143 (SD: AUD 77,733; median: AUD 50,000) which is comparable to the national average of AUD 60,600 (AUD 1164.60 weekly [33]). Many (38%) had a university degree or a certificate or diploma (34%; Table 1). This is comparable with the sample of other online surveys which tend to undertaken by better educated people than those participating in other survey modes [34,35]. Most of the respondents (64%) lived in the most populated states of Australia (Victoria, New South Wales and Queensland; Table 1).

Most respondents were in full-time positions (61%), 34% part-time and 5% employed on a casual basis. Thirty-five percent worked irregular hours (e.g., shift work or “fly-in/fly-out”), the other 65% regular hours. Most respondents (64%) worked in the private industry, 24% in the public sector and 12% were self-employed across different sectors. The majority (62%) of respondents reported that they were in good health and very few self-rated their health as poor (3%; Table 1). We did not detect significant differences across the samples from the two survey stages for core demographic parameters (Table S3).

### 3.2. Level of Heat Stress

A quarter of all 1719 respondents said they never felt stressed by heat at work. Most felt heat stressed sometimes (30%) or rarely (28%; Table 2). Ten percent said they were often heat stressed and 7% very often. In subsequent analyses, the last two categories were combined to 17% feeling often or very often heat stressed. Those who considered themselves to have excellent or good health were more likely to be never, and less likely to be often or very often, heat stressed than those with fair or poor health (KW = 23.51; *p* < 0.001). The level of heat stress was not affected by respondent’s age or gender (control variables), and also not by the state they lived in (environment).

The higher the score of physical exertion (F = 56.22; *p* < 0.001), the higher respondents’ heat stress levels (Figure S1a). Respondents’ spending hardly any working time outside were more likely never or rarely to be heat stressed (KW = 64.81; *p* < 0.001) (Respondents who said they were often or very often heat stressed were significantly more likely to work in jobs that required them to spend about three-quarters of their work time outside, but not more likely to work outside “almost all the time“ (Figure S1b).

The level of heat stress did not differ greatly across the sectors (Table 2). Only people employed in education and finance were more likely never to be heat stressed than being rarely, sometimes or often heat stressed (KW = 17.42; *p* < 0.001). People in the education sector were the most likely to report never feeling heat stressed (35.2%) while those in the manufacturing sector (14.4%) share of people were least likely to. Health Care and Social Assistance (13%) had lowest share of people often and very often heat stressed and Arts, Recreation, Tourism, Food, Hospitality (25%) the highest.

### 3.3. Symptoms of Heat Stress

Fatigue and headaches were the most frequently mentioned symptom associated with heat stress (both about 56%; Table 3). The symptoms differed significantly across the level of heat stress with seizure, fainting, nausea, skin rashes and irritability more likely to have been mentioned by people who often or very often felt heat stressed than by people who rarely or sometimes felt heat stressed. Dizziness and headaches were more likely to be reported as the level of heat stress went up. Fatigue and irritability occurred less among people who were rarely heat stressed.

### 3.4. Immediate Relief Measures

Bivariate analysis showed that, of the three immediate relief measures, only resting was affected by the level of heat stress people experienced (KW = 11.61; *p* = 0.003) with those rarely heat stressed less likely to rest (Table 4). The long-term relief strategy of changing jobs, on the other hand, was highly affected by how heat stressed people said they were (KW = 113.46; *p* < 0.001). Of those often or very often heat stressed, 27% said that they would eventually change jobs because of heat at their workplace. For those sometimes heat stressed, this was 8% and for those rarely heat stressed only 2% (Table 4).

The heat relief measures taken were little affected by the sector in which respondents were working; not at all for hydrating and resting and only slightly for cooling with those working in the education and finance sector most likely cope with heat stress by cooling (Table 4). Respondents working in “Arts, Recreation, Tourism, Food, Hospitality” were the most likely to intend to change their jobs because of heat stress, while those working in “Health Care and Social Assistance” were the least likely to do so (KW = 28.94; *p* < 0.001).

#### 3.4.1. Hydration

**Figure 1:**
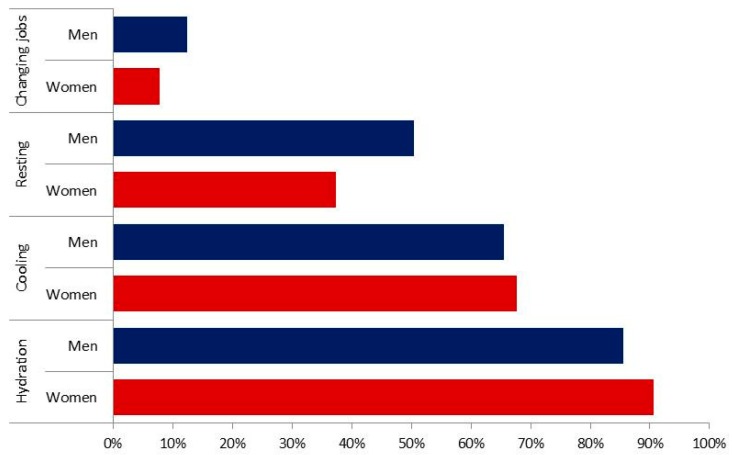
Percentage of heat stressed respondents (N = 1285) adopting specific heat relief measures—by gender.

Eighty-eight percent of the respondents who reported heat stress used hydration as a relief measure with only slight differences between genders (Figure 1). Men were 38% ((0.62/(1 + 0.62)) less likely to hydrate than women (Table 5), and no other variables significantly explained any variation in this outcome.

#### 3.4.2. Cooling

Sixty-seven percent of the respondents said that they cooled down when feeling heat stressed. Education was one of the significant determinants of cooling behaviour as a response to heat stress (Table 5). In fact, those with university degree were about 60% (1.46/(1 + 1.46)) more likely to cool down than those with lower levels of education. The higher the physical demand of respondent jobs, the less likely they were to cool down and those who reported of being of poor health were 85% more likely to cool down than those with better health ((5.71/(1 + 5.71)).

#### 3.4.3. Resting

Forty-four percent of heat stressed respondents rested when feeling heat stressed. Male respondents were 61% more likely to rest than females ((1.59/(1 + 1.59)) and those with poor health 67% more likely than those with fair and good health ((2.04/(1 + 2.04)). Those in physically demanding jobs were more likely to rest, as were those who were spending more of their worktime outside. Contrary to the bivariate analysis, the level of heat stress was not significant in the multivariate logit model (Table 5).

#### 3.4.4. Combination of Immediate Relief Measures

About a third of respondents (31%) reported that they only adopted one of the three relief measures (cooling, hydrating, resting), 39% undertook two measures and 30% all three. Almost all (89%) who said they aimed to cool down also hydrated, and also 89% of those who rested also hydrated. In fact, 99% of those who adopted two of the measures included hydration, i.e., the combination cooling and resting was very rare. Thus, 59% of all of the heat stressed respondents coped using a combination of cooling and hydrating. Sixty-three percent of respondents only mentioned hydrating as single measure, 22% only cooling and 15% would only rest.

### 3.5. Changing Jobs as Long-Term Adaptation Measure

Only 7% of respondents who felt heat stressed at work would change their jobs because of that the heat. This response depended on the level of reported heat stress with those often or very often heat stressed being 92% (11.88/(1 + 11.88)) more likely to intend to change their jobs than those rarely feeling heat stressed (Table 5). Those who reported they were heat stressed sometimes were 76% more likely to intend to change their jobs (3.23/(1 + 3.23)). While the saturated model showed a significant and positive interaction effect between “Male” and “Physical exertion” (see Table S8), the best model did not support inclusion of this interaction term.

### 3.6. Organizational Heat Stress Relief Management

Only a few respondents (8%) did not see a need for heat prevention and relief measures at their workplace (Table 6). The majority (41%) thought that they were handled well and 34% that they were ok. Seventeen percent of respondents thought that heat stress was being handled poorly at their workplace. Respondents with a high degree of heat stress were more likely to think that they were handled poorly than those sometimes stressed and those rarely stressed (29% vs. 20% vs. 7%; *p* < 0.001). Those rarely stressed were, in return, more likely to say that there was no need for such measures at their workplace and that they were handled well (Table 6).

Self-employed respondents were less likely (8.5%) to describe heat relief management as poor than those in the private (17.5%) and public (19.5%) sectors (*p* < 0.005). Self-employed respondents were also more likely to see no need for these measures (*p* < 0.01) (Table 6). There were no significant differences in what people thought about their workplace heat relief plans and the sectors they worked in.

## 4. Discussion

### 4.1. Heat Stress Symptoms and Intensity

Most heat stressed respondents experienced light symptoms, mostly fatigue and headaches, as shown in previous studies from Australia [36,37] and Europe [38,39]. The more severe symptoms such as dizziness, skin rashes, confusion and nausea were mainly reported by those with high levels of heat stress.

It was surprising that three of the four control variables (gender, age and location) had no significant impact on the intensity or level of heat stress. We would have expected that respondents from the cooler regions (e.g., Tasmania) would be less likely to be often heat stressed than those in hotter jurisdictions such as Queensland and the Northern Territory. Even when the locations where people lived were separated into the tropical parts of northern Australia, characterized by high temperatures throughout the year and high humidity during large parts of the year (wet season), and southern Australia, we could detect no significant difference in level of heat stress, contrary to expectations [40]. Only one control variable, self-reported health, had a significant (*p* < 0.001) impact on respondents’ heat stress levels with those in better health less likely to say they were often or very often heat stressed than those in poorer health. This finding is consistent with studies showing those in poor health (i.e., with pre-existing illnesses) are more likely to suffer heat stress-related illnesses during heat waves [9,36,41,42] and that those with pre-existing illnesses are most likely to die from exposure to elevated ambient temperatures [30,31].

It was also surprising that respondents employed in outdoor and labour intensive industries were not more likely to be often or sometimes heat stressed (Table 2), just less likely to be rarely heat stressed. While it is not the industry per se that affects heat stress levels, we found that the level of heat stress was positively correlated with the amount of physical exertion at work (see Figure S2). While this was an expected result [21,43], the relationship between working time spent outside and heat stress levels was less pronounced. Those spending almost all of their working time outside seemed to have developed some degree of acclimatisation.

### 4.2. Immediate Heat Relief Measures Taken at Work

In line with other research from Australia [7,36,44,45] and Europe [38], hydrating was the most common heat relief measure with nearly 90% of respondents employing it, often in combination with either cooling or resting. Drinking is very important for avoiding dehydration if a substantial volume of fluid is lost through sweating. Not surprisingly, given this homogenous response to hydrating, we did find many factors that would explain the little variation. However, there is a gender effect: men were slightly less likely to hydrate than women. Men were also more likely to rest. This pattern might depend on the type of job men have and whether jobs are conducted in a controlled weather environment (air conditioned) or outside and on the type of job and its physical intensity, with both factors positively related to the likelihood of resting.

People in physically demanding jobs are not only more likely to rest, they are also less likely to cool down. This is likely to be important for labour productivity if heat stress results in a significant proportion of peoples’ work time is spent resting. Another study from Australia showed that losses from reduced labour productivity exceed AUD 7 billion per year [19]. While people involved in physically demanding jobs prefer to rest, it is unsure what kind of resting environments are available for workers. For instance, while resting and cooling off in a controlled environment, such as with air conditioners, can be useful, the efficacy of fans as cooling devices are more complex and have mixed effects on elderly [46].

It was surprising that the heat relief measures taken were so similar across different sectors, at least for hydrating and resting. We would have expected respondents working in labour intensive sectors or industries to be resting more often. At least this has been the overall conclusion of previous research [4,7]. In fact, each sector appears to be affected by people self-pacing their work and resting when heat stressed and thereby reducing their productivity. Cooling was slightly affected by the sector, with those in a low labour intensive sector (education and finance) being more likely to cool. However, we think that these people simply refer to passive cooling which they do by working in an air-conditioned environment. Those more likely to cool also were better educated, strengthening our assumptions that these people might work in office jobs and cool in air-conditioning to relieve heat stress.

We would have expected that any of the immediate relief measure choices depended on the climate in which people lived. In humid hot weather, it is difficult to sweat and cool off, unlike in dry hot weather. Rehydration may not be helpful in very humid hot weather and in some cases, excessive water consumption can result in hyponatraemia, a condition with low sodium levels in the blood [47]. However, the location was not significant for any of the measures. The fact that respondents from the hotter parts of Australia (e.g., the tropical North) do not rest or cool more than respondents from other parts could be another indication of acclimatisation.

We confirmed that those with poorer health need to rest more, potentially contributing to delays in work chains and productivity loss. As suggested elsewhere [5], health screening for such jobs or areas with usually high average temperatures can help to minimize the risk of the health of unhealthy people deteriorating further from additional heat stress.

As for combinations of immediate relief measures, about a third of respondents hydrated, rested and cooled at the same time and even fewer would rest and cool together. This implies that very few people would use active cooling measures such as arm immersion cooling and ice slurry because these also require resting at the same time. Most people would just hydrate (drink) while continuing to work or hydrate and cool (passively, i.e., working in air-conditioned environments) or hydrate and rest. However, active cooling, which also requires resting, is the most effective form of mitigating heat stress, particularly in warm and humid climates, as has been shown for fire fighters [48], army personnel [49] and athletes [50]. People in labour intensive jobs should do the same, as resting in very hot conditions without cooling at the same time does not bring down core temperature [50].

### 4.3. Changing Jobs as an Extreme Form of Adaptation

Changing jobs, as the most extreme form of adapting to heat stress at work and a long-term strategy that involves some planning and a significant change in life, is the only measure that was significantly correlated with the level of heat stress and was mainly being considered as an option for those sometimes and often heat stressed. We found that those exerting physical labour at their workplace were more likely to change jobs. In hot (and humid) regions, such as tropical Australia and many remote inland locations, changing jobs entails changing location. This can exacerbate the already high turn-over rates in some jobs and the problem of attracting new labour into these remote areas. Highly mobile workers such as fly-in/fly-out (FIFO) staff common in mining companies operating in remote Australia may be travelling across climate zones and may not follow adequate heat relief measures sensitive to the climate in which they are operating, potentially leading to heat exhaustion and eventually quitting the job.

### 4.4. Acclimatisation

For a relatively hot continent such as Australia acclimatisation will be very important as average temperatures increase. A healthy heat-acclimatised person can tolerate extended exposure to any weather-related heat stress [51], reducing the health and economic burden of heat. Some of our results can be explained by acclimatisation by some of the respondents. For example, the lack of a strong link between time working outside and heat stress points to people spending a lot of their work time outside have become acclimatised. Heat acclimatisation is most effective when directly exposed to hot environments outside [52]. In sport science, it has been shown that maximum level of acclimatisation can be achieved by training in the outside heat for between one and two weeks [52].

Another reason acclimatisation might explain some of our results is that respondents from the hotter parts of Australia do not rest or cool more than respondents from cooler regions. By living in a constantly hot environment, they might have gained long-term acclimatisation, and do not need additional heat relief strategies. Therefore, health and safety guidelines at workplaces should be targeted separately for those acclimatised (after they have been in a hot environment for two weeks) and those not acclimatised. This would increase productivity as those acclimatised can work more. On the other hand, those new to a hot environment must be made aware of the process of acclimatisation and so they self-pace their work, taking regular heat relief measures when appropriate for both the conditions and their degree of acclimatisation. Other ways of ensuring the health and safety of work in hot places is to select workers specifically for a particular occupation who are likely to be heat adapted. This might make the recruitment process more expensive and time consuming but is likely to result in lower labour productivity loss, lower accident rates, higher retention rates of workers and more satisfied workers. Moreover, it begs the question why, in some hot and humid parts of Australia, more people from a similar climate are not recruited, but instead a large part of the labour force are FIFO workers from cooler parts of the country.

Acclimatisation decays rapidly [53] but the capacity to acclimatise is retained for up to two months and can be regained within two days then [54]. Because it occurs not only through morphological and chemical adjustments but through epigenetic mechanisms [50,54]. However, this means, that FIFO workers, military personnel and other civilians who increasingly have to relocate to different climate zones within a short period of time, are constantly gaining and losing acclimatisation, which must affect their productivity.

### 4.5. Satisfaction with Heat Management Implementation

The percentage of respondents who said that heat stress was handled poorly at their work was low (17%). This is a proportion that should not be neglected, particularly since this was perceived by people who were often heat stressed. Previous research [7] showed that people in southern Australia in physical demanding jobs were particularly satisfied with their workplaces’ preventive measures. We did not detect any of those effects. Thus, the effects of heat on workforce turnover are not associated with equity theory [55] but to personal individual discomfort.

### 4.6. Survey Limitations and Future Research Needs

A potential limitation of this study was that the data were obtained from self-reported measures, commonly applied in survey research methodology [56] and so the results should be used with some caution in the interpretation. Our sample is quite large, which helps alleviate biases arising from small self-selected samples. To further verify the robustness of our sample, we conducted the survey in two stages. We did not detect any differences among core demographic parameters, making it unlikely that there was significant bias from non-completion of the survey. There were also no differences in heat relief measures, other than for drinking with participants in survey Wave 2 slightly more likely to drink (see Table S3). Heat stress was also not significantly different across the two stages, implying some degree of validation of the results through replication.

The research was further limited to very general relief measures when more defined ones are already being deployed, such as changing schedules to working early morning or late afternoons to minimize extreme heat exposure. Other administrative controls include shift rotation and additional relief workers or wearing circulating air suits [57]. Such practices, however, are most common in labour intensive and outdoor industries as well as to jobs in very hot environments (e.g., in a bakery) and no research has yet been done on whether the general public applies these more detailed measures. The survey would also have benefited had we asked in more detail how some of the relief measures were being used, for instance, asking people the frequencies with which they drink, how much resting they do and what else they did while resting, to assess effective combinations of measures.

Finally, the survey was limited to personal immediate measures (hydration, cooling and resting) and personal long-term heat relief (changing jobs) and we did not go into much detail about organizational measures (other than asking if respondents were satisfied with them). More research is needed in the assessment of detailed organizational measures in combination with personal ones.

## 5. Conclusions

We found that people in labour intensive and outdoor work sectors were not more likely to be often or sometimes stressed than those in non-vulnerable jobs. This indicates that other sectors also need heat management and prevention plans. More research specific to climate zones could also help employers develop heat relief guidelines for highly mobile labour force that need to relocate between different climate zones within a short period of time. Labour productivity loss from heat stress and associated illness in many of the social service providing industries will be a high cost to the economy.

High risk groups of people who are likely to suffer from heat stress need to be identified and targeted across all sectors, including those working in office-based sectors. We found some indication that some respondents were heat-acclimatised. Who these are needs to be better investigated, as they are the least affected by heat at work, and therefore should be the preferred work force for specific climatically distinct regions (e.g., extreme dry regions; and extremely hot and humid regions) or employment sectors. There needs to be guidelines for heat relief measures and work health safety plans specific to varying climates and specifically vulnerable populations. These guidelines will need to be evidence based and will need to consider future climatic change.

## Figures and Tables

**Table 1:** Sample characteristics (N = 1719).

Personal Characteristic	%	N	Work Related Characteristic	%	N
Gender			Work method		
Male	52	903	Regular hours (“9 to 5”)	65	1120
Level of education			Work time spent outside		
University degree	38	657	Almost none (less than 10% of time)	68	1172
Certificate or Diploma	34	588	About a quarter of work time	14	244
Completed high school	14	247	About half of work time	8	132
Completed Years 11 or 10	11	194	About three-quarters of work time	4	63
Year 9 or below	2	33	Almost all (more than 90% of time)	6	108
Health status			Workload		
Excellent health	16	276	Full-time	61	1055
Good health	62	1062	Part-time	34	579
Fair health	19	326	Casual	5	85
Poor health	3	55			
State			Employee		
Victoria (VIC)	24	406	Private sector	64	1095
New South Wales (NSW)	23	397	Public sector	24	415
Queensland (QLD)	17	297	Self-employed	12	209
Western Australia (WA)	13	224			
South Australia (SA)	9	151			
Australian Capital Territory (ACT)	6	102			
Tasmania (TAS)	5	89			
Northern Territory (NT)	3	53			

**Table 2:** Degree of heat stress varying across different variables (N = 1719)—results from bivariate analyses.

Variable	% of Respondents	Never Stressed	Rarely Stressed	Sometimes Stressed	Often and Very Often Stressed
Total number of respondents (%)		434 (25.2)	473 (27.5)	524 (30.5)	288 (16.8)
Category: Outdoor industries					
Agriculture, Forestry, Fishing, Gardening, Pastoralism, Army, Power, Waste, Mining	0.8	1.4 ^a^	0.6 ^a^	0.3 ^a^	1.0 ^a^
Construction	6.6	5.8 ^a^	6.8 ^a^	6.7 ^a^	7.3 ^a^
Category: Labour intensive industries					
Manufacturing	4.8	2.8 ^a^	6.1 ^a^	5.5 ^a^	4.5 ^a^
Transport, Postal and Warehousing	5.4	4.4 ^a^	4.9 ^a^	5.7 ^a^	7.3 ^a^
Category: Indoor industries					
Arts, Recreation, Tourism, Food, Hospitality	8.8	6.5 ^a^	8.2 ^a^	9.0 ^a^	13.1 ^a^
Health Care and Social Assistance	14.3	13.3 ^a^	16.1 ^a^	15.3 ^a^	11.1 ^a^
Education/Finance	14.7	20.5 ^a^	11.2 ^b^	14.3 ^b^	12.5 ^b^
Professional, Scientific and Technical Services	12.9	15.2 ^a^	15.0 ^a^	9.9 ^a^	11.1 ^a^
Public Administration and Safety	12.0	13.1 ^a^	12.3 ^a^	11.0 ^a^	118 ^a^
Retail/Wholesale	14.1	13.1 ^a^	13.5 ^a^	14.9 ^a^	15.2 ^a^

Note: If two variables within a category have different superscript letters (“a” and “b”) the level of heat stress is significantly different among those (*p* < 0.05).

**Table 3:** Self-reported symptoms of heat stress in the workplace of those respondents reported heat stress (%)—by degree of heat stress (N = 1285).

Symptoms	% of Respondents with Symptom	Rarely Stressed	Sometimes Stressed	Often and Very Often Stressed	Test Statistics (KW)
Fatigue	56.1	48.6 ^a^	59.9 ^b^	61.5 ^b^	17.18 ***
Headache	55.8	47.1 ^a^	57.1 ^b^	67.7 ^c^	31.23 ***
Irritability	26.2	21.1 ^a^	26.9 ^ab^	33.3 ^b^	13.95 ***
Skin rash	23.5	17.1 ^a^	23.3 ^a^	34.4 ^b^	29.63 ***
Dizziness	19.0	10.1 ^a^	18.9 ^b^	33.7 ^c^	64.40 ***
Confusion	6.8	3.6 ^a^	5.7 ^a^	13.8 ^b^	31.56 ***
Nausea	5.8	3.2 ^a^	5.3 ^a^	11.1 ^b^	20.90 ***
Fainting	4.7	3.4 ^a^	3.4 ^a^	9.3 ^b^	17.57 ***
Seizure	0.7	0.0 ^a^	0.6 ^a^	2.1 ^b^	11.37 **

Note: Same superscript letters (“a”, “b” and “c”) denote that the symptoms are not significantly different across the heat stress levels; different superscript letters that the symptom vary significantly with the level of heat stress; the superscript “ab” denotes that the symptom is not statistically different to the ones with “a” and “b”. KW = Kruskal–Wallis H test statistic, *** = 1% significance level; ** = 5% significance level.

**Table 4:** Percentage of heat stressed respondents using different relief measures—by level of heat stress and work sector (N = 1285).

Level of Heat Stress and Work Sector	% of Respondents Hydrating	% of Respondents Cooling	% of Respondents Resting	% Intending To Change Jobs
Leve of heat stress				
Rarely	88.4 ^a^	66.6 ^a^	38.1 ^a^	2.3 ^a^
Sometimes	89.1 ^a^	67.9 ^a^	48.3 ^b^	7.8 ^b^
Often and very often	85.1 ^a^	63.9 ^a^	46.9 ^b^	26.5 ^c^
Sector				
Agriculture, Forestry, Fishing, Landscape, Gardening, Pastoralism, Army, Power, Waste, Mining	87.1 ^a^	55.3 ^ab^	54.1 ^a^	6.4 ^ab^
Arts, Recreation, Tourism, Food, Hospitality	94.4 ^a^	55.6 ^a^	37.1 ^a^	20.5 ^c^
Construction	85.2 ^a^	65.9 ^ab^	52.2 ^a^	10.7 ^ac^
Education/Finance	87.8 ^a^	73.8 ^b^	46.3 ^a^	8.1 ^ab^
Health Care and Social Assistance	88.3 ^a^	70.2 ^ab^	38.3 ^a^	5.7 ^a^
Manufacturing	80.3 ^a^	62.0 ^ab^	47.9 ^a^	7.6 ^ac^
Professional, Scientific and Technical Services	82.6 ^a^	69.7 ^ab^	41.9 ^a^	7.0 ^ab^
Public Administration and Safety	86.0 ^a^	68.7 ^ab^	45.3 ^a^	9.0 ^ac^
Retail/Wholesale	91.9 ^a^	66.1 ^ab^	43.5 ^a^	16.4 ^bc^
Transport, Postal and Warehousing	93.2 ^a^	67.6 ^ab^	45.9 ^a^	9.0 ^ac^

Note: Same superscript letters denote that the likelihood of respondents applying a relief measure did not differ across the heat stress levels or the work sector; different superscript letters that the relief measures are taken up more or less often across respondents with different heat stress levels or in different sectors; the superscript “ab” denotes that the relief measure is not statistically different to the ones with “a” and “b; “ac” that it is not different to the ones with “a” and “c”, and “bc” that it is not different to the ones with “b” and “c”

**Table 5:** Results of separate logit models for each heat stress relief/adaptation measure, model coefficients (Coeff), odds ratio (OR), and 95% confidence interval (lower and upper limit).

Variables	Coeff	OR	5%	95%	*p*-Value
Hydration					
Constant	2.265	9.63	7.40	12.79	<0.001
Male	−0.485	0.62	0.43	0.88	0.006
Cooling					
Constant	0.931	2.54	1.83	3.54	<0.001
University degree	0.380	1.46	1.13	1.89	0.004
Physical exertion	−0.107	0.90	0.86	0.94	<0.001
Poor health	1.741	5.71	2.28	19.12	<0.001
Resting					
Constant	−1.229	0.29	0.22	0.39	0.001
Male	0.467	1.59	1.27	2.01	<0.001
Poor health	0.715	2.04	1.11	3.85	0.023
Physical exertion	0.100	1.10	1.06	1.16	<0.001
Time working outside	0.119	1.13	1.02	1.24	0.019
Changing jobs					
Constant	−4.497	0.01	0.001	0.02	<0.001
Physical exertion	0.157	1.17	1.08	1.27	<0.001
Sometimes heat stressed	1.171	3.23	1.65	6.93	0.001
Often or very often heat stressed	2.475	11.88	6.22	25.13	<0.001

This note can be deleted. It referred to a variable no longer used.

**Table 6:** Opinion about how heat stress prevention and relief is managed at respondents’ workplace (% of respondents)—by degree of heat stress and employee (N = 1285).

Level of Heat Stress and Employee	Handled Well	Handled OK	Handled Poorly	No Need for It
% of respondents	40.9	34.2	16.9	8.0
Level of heat stress				
Rarely stressed	48.6 ^a^	33.0 ^ab^	6.8 ^a^	11.6 ^a^
Sometimes stressed	35.9 ^b^	37.8 ^a^	19.7 ^b^	6.7 ^b^
Often and very often stressed	37.5 ^b^	29.5 ^b^	28.5 ^c^	4.5 ^b^
Employee				
Employed in public sector	43.3 ^a^	30.8 ^a^	19.5 ^a^	6.2 ^a^
Employed in private sector	39.8 ^a^	35.0 ^a^	17.5 ^a^	7.6 ^a^
Self-employed	42.1 ^a^	36.0 ^a^	8.5 ^b^	13.4 ^b^

Note: Same superscript letters (“a”, “b” and “c”) denote that the opinions are not significantly different across the heat stress levels or across the employee; different superscript letters that the opinions vary significantly with the level of heat stress or employee; the superscript “ab” denotes that the opinion is not statistically different to the ones with “a” and “b”.

## Supplementary Materials

The following are available online at http://www.mdpi.com/1660-4601/15/3/401/s1, Figure S1, The impacts of physical exertion of work (a) and time spent outside (b) on respondents’ heat stress levels (N = 1719), Figure S2, Heat relief measures taken by heat stressed respondents (N = 1719) at work by their levels of physical exertion at work, Table S1, Number of invited survey participants, drop-out and response rates for the two survey waves in May (Wave 1) and October 2014 (Wave 2), Table S2, Variables included in logit models as determinants of heat relief measures, Table S3, Differences in core demographics and heat relief measures of respondents in survey Wave 1 and Wave 2, Table S4, Correlation matrix of independent variables regarding demographic and employment background, and heat stress (as described in Table S2), Table S5, Results of saturated logit models with interactions between core demography variables—Hydration, Table S6, Results of saturated logit models with interactions between core demography variables—Cooling, Table S7, Results of saturated logit models with interactions between core demography variables—Resting, Table S8, Results of saturated logit models with interactions between core demography variables—Changing job.
