# Supplementary material for: Exploring Heat Stress Relief Measures among the Australian Labour Force
Source: Int J Environ Res Public Health. 2018 Feb 26;15(3):401. doi: 10.3390/ijerph15030401 (PMC5876946; doi:10.3390/ijerph15030401)
Supplement: Supplementary file 1 [file ijerph-15-00401-s001.pdf]

Supplementary materials

Table S1. Number of invited survey participants, drop-out and response rates for the two survey waves in May (Wave 1) and October 2014 (Wave 2).

Survey Responses	Total	Wave 1	Wave 2
Invited respondents	9405	4912	4493
Started survey (%)	2087	871	1216
Dropped-out (%)	210	72	138
Excluded (%)	158	51	107
Final sample	1719	748	971
Response rate	18.3%	15.2%	21.6%

Table S2. Variables included in logit models as determinants of heat relief measures.

Category	Variables	Coding
Heat stress	Heat stress	3 variables, dummy coded:<br>- rarely<br>- sometimes<br>- often/very often
Workplace	Workload	Dummy coded: full-time (1) / casual or part-time (0)
	Time spent outside while working	1 variable coded from 0 to 4:<br>- Almost no time (0)<br>- About a quarter of work time (1)<br>- About half of work time (2)<br>- About three-quarters of work time (3)<br>- Almost all time (4)
	Physical exertion	1 variable coded from 1 (very low) to 10 (very high)
	Satisfaction with heat relief management at work	4 variables, dummy coded:<br>- handled well<br>- handled ok<br>- handled poorly<br>- no need for heat management at work
Environment	Location (State/Territory)	8 variables, dummy coded:<br>- Victoria (VIC)<br>- New South Wales (NSW)<br>- Queensland (QLD)<br>- South Australia (SA)<br>- Western Australia (WA)<br>- Tasmania (TAS)<br>- Australian Capital Territory (ACT)<br>- Northern Territory (NT)
Control variables	Age	continuous
	Income	continuous
	Gender	Dummy coded: male (1) / female (0)
	Level of education	5 variables, dummy coded:<br>- University degree<br>- Certificate/Diploma<br>- Completed high school<br>- Completed Years 11 or 10<br>- Year 9 or below
	Health status	Dummy coded: poor health (1) / good health (0)

Note: All perception variables were coded: 1 (Strongly disagree), 2 (Disagree), 3 (Agree), 4 (Strongly agree) to the respective statements.

Table S3. Differences in core demographics and heat relief measures of respondents in survey wave 1 and wave 2.

Sample Characteristics	Wave 1	Wave 2	Difference
Number of respondents	748	971	
Male (%)[+]	381 (50.9%)	522 (53.8%)	χ^2 = 1.351; p = 0.245
Age (years)	40.5 (SD: 12.5)	40.6 (SD: 11.9)	F = 0.070; p = 0.791
Income (Mean)	61,630 (SD: 78,898)	60,769 (SD: 76,863)	F = 0.052; p = 0.820
Stressed by heat	557 (74.5%)	728 (75.0%)	χ^2 = 0.058; p = 0.810
Work full-time	450 (60.1%)	605 (62.3%)	χ^2 = 0.821; p = 0.365
Cooling	369 (66.2%)	486 (66.8%)	χ^2 = 0.037; p = 0.848
Resting	241 (43.3%)	327 (44.9%)	χ^2 = 0.348; p = 0.555
Drinking	473 (84.9%)	657 (90.3%)	χ^2 = 8.445; p = 0.004
Thought that heat relief management at work was handled well	221 (39.7%)	305 (41.9%)	χ^2 = 0.932; p = 0.334

Note: [+] Exactly the same numbers of female and male respondents were invited; it happened that more men choose to participate in the survey during wave 2.

Table S4. Correlation matrix of independent variables regarding demographic and employment background, and heat stress (as described in Table S2).

Sample Characteristics	Time Spent Outside	Physical Exertion	Age	Income	Male	University Degree	Poor Health	Sometimes Heat Stressed	Often or very often Heat Stressed
Fulltime employment	−0.013	−0.067	0.049	0.284	0.277	0.083	−0.080	−0.064	0.036
Time spent outside	1	0.339	0.095	−0.005	0.188	−0.142	0.042	0.107	0.095
Physical exertion		1	0.025	−0.085	0.046	−0.146	−0.001	0.095	0.231
Age			1	0.094	0.079	−0.155	0.047	0.023	−0.014
Income				1	0.153	0.089	−0.048	−0.021	−0.014
Male					1	−0.017	−0.019	0.004	−0.004
University degree						1	−0.055	−0.053	−0.013
Poor health							1	0.023	0.087
Sometimes heat stressed								1	−0.297
Often or very often heat stressed									1

Table S5. Results of saturated logit models with interactions between core demography variables—Hydration.

Variables	Coefficient	Standard Error	*p*-Value
Constant	1.986	0.458	<0.0001
Age	−0.007	0.007	0.3618
Poor Health	7.272	5.725	0.2040
Age * Poor Health	−0.118	0.103	0.2535
Physical exertion	0.094	0.054	0.0845
Male	−0.036	0.372	0.9237
Physical exertion * Male	−0.103	0.069	0.1339
Time working outside	0.076	0.079	0.3338
University degree	−0.003	0.197	0.9868
Fulltime	−0.219	0.213	0.3032
Income	0.004	0.003	0.1517
Sometimes heat stressed	0.014	0.204	0.9454
Often or very often heat stressed	−0.396	0.231	0.0872

Note: * = indicating interaction between the two variables.the symbol "*".

Table S6. Results of saturated logit models with interactions between core demography variables—Cooling.

Variables	Coefficient	Standard Error	*p*-Value
Constant	0.916	0.314	0.0035
Age	0.0002	0.005	0.9663
Poor Health	1.618	2.060	0.4321
Age * Poor Health	0.003	0.044	0.9396
Physical exertion	−0.100	0.034	0.0037
Male	−0.082	0.275	0.7646
Physical exertion * Male	0.006	0.047	0.9064
Time working outside	−0.056	0.052	0.2855
University degree	0.374	0.133	0.0048
Fulltime	0.006	0.136	0.9671
Income (in thousands‘~~000~~)	0.001	0.001	0.4134
Sometimes heat stressed	0.128	0.139	0.3577
Often or very often heat stressed	0.016	0.167	0.9240

Note: ~~Please confirm whether "'000" is correct. A~~* = indicating interaction between the two variables.~~nd add the explanation for the symbol "*".~~

Table S7. Results of saturated logit models with interactions between core demography variables—Resting.

Variables	Coefficient	Standard Error	*p*-Value
Constant	−1.321	0.310	<0.0001
Age	−0.007	0.005	0.1696
Poor Health	−0.171	1.266	0.8927
Age * Poor Health	0.020	0.027	0.4714
Physical exertion	0.105	0.034	0.0018
Male	0.559	0.260	0.0315
Physical exertion * Male	−0.009	0.045	0.8421
Time working outside	0.116	0.051	0.0247
University degree	0.267	0.131	0.0422
Fulltime	−0.194	0.130	0.1366
Income (in thousands‘000)	0.001	0.001	0.1057
Sometimes heat stressed	0.318	0.133	0.0171
Often or very often heat stressed	0.145	0.162	0.3704

* = indicating interaction between the two variables.

Table S8. Results of saturated logit models with interactions between core demography variables—Changing job.

Variables	Coefficient	Standard Error	*p*-Value
Constant	−3.153	0.621	<0.0001
Age	−0.023	0.009	0.0122
Poor Health	−0.236	1.618	0.8843
Age * Poor Health	0.024	0.035	0.5000
Physical exertion	0.055	0.064	0.3890
Male	−0.381	0.554	0.4908
Physical exertion * Male	0.188	0.086	0.0290
Time working outside	−0.032	0.084	0.7057
University degree	0.175	0.234	0.4555
Fulltime	−0.489	0.242	0.0433
Income (in thousands~~'000~~)	−0.002	0.002	0.3478
Sometimes heat stressed	1.132	0.367	0.0020
Often or very often heat stressed	2.494	0.357	<0.0001

* = indicating interaction between the two variables.

(a)

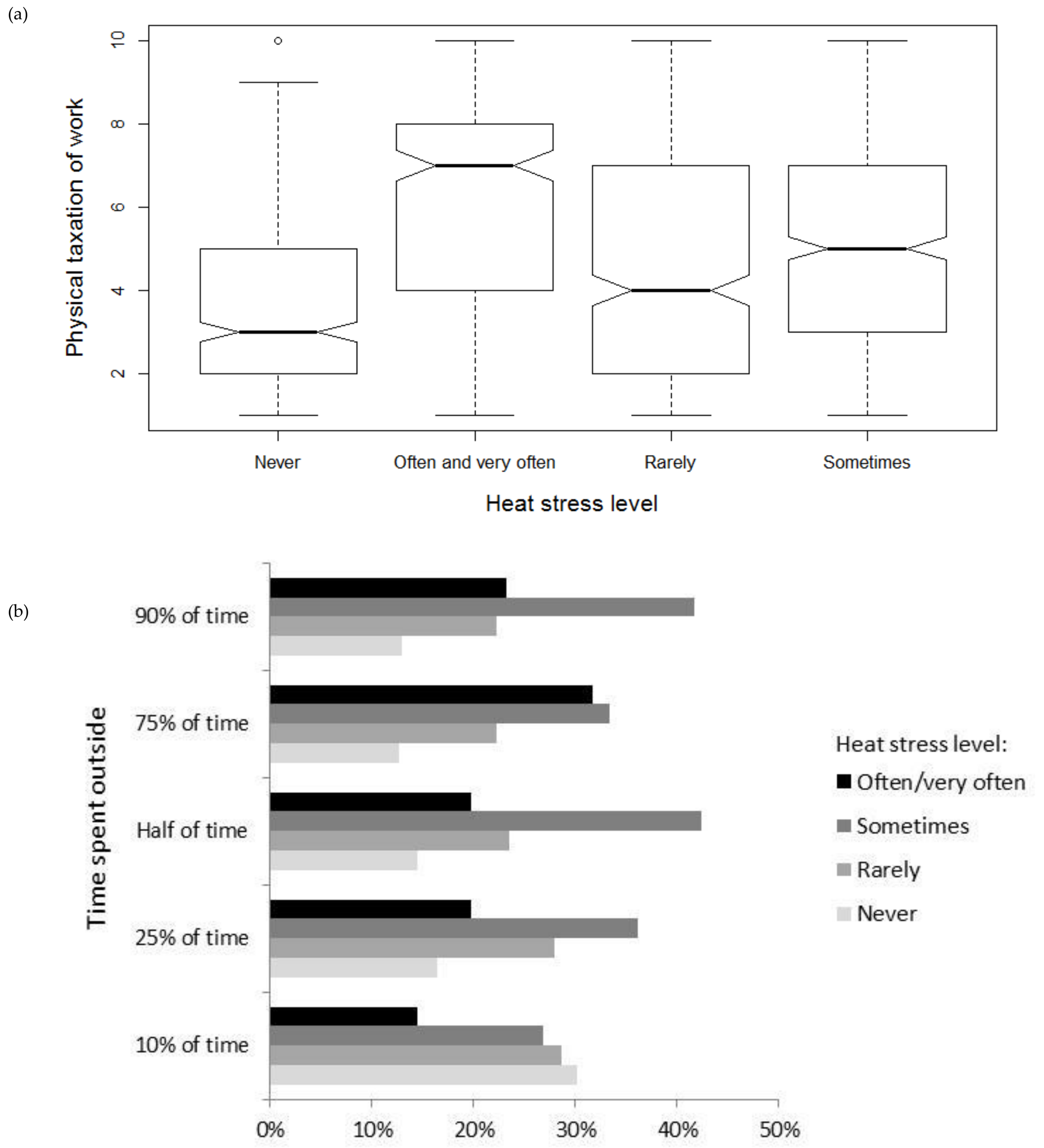

Figure S1. The impacts of physical exertion of work (**a**) and time spent outside (**b**) and on respondents' heat stress levels (N = 1719). (Please explain the different colour in Figure S1a, such as: red, grey).

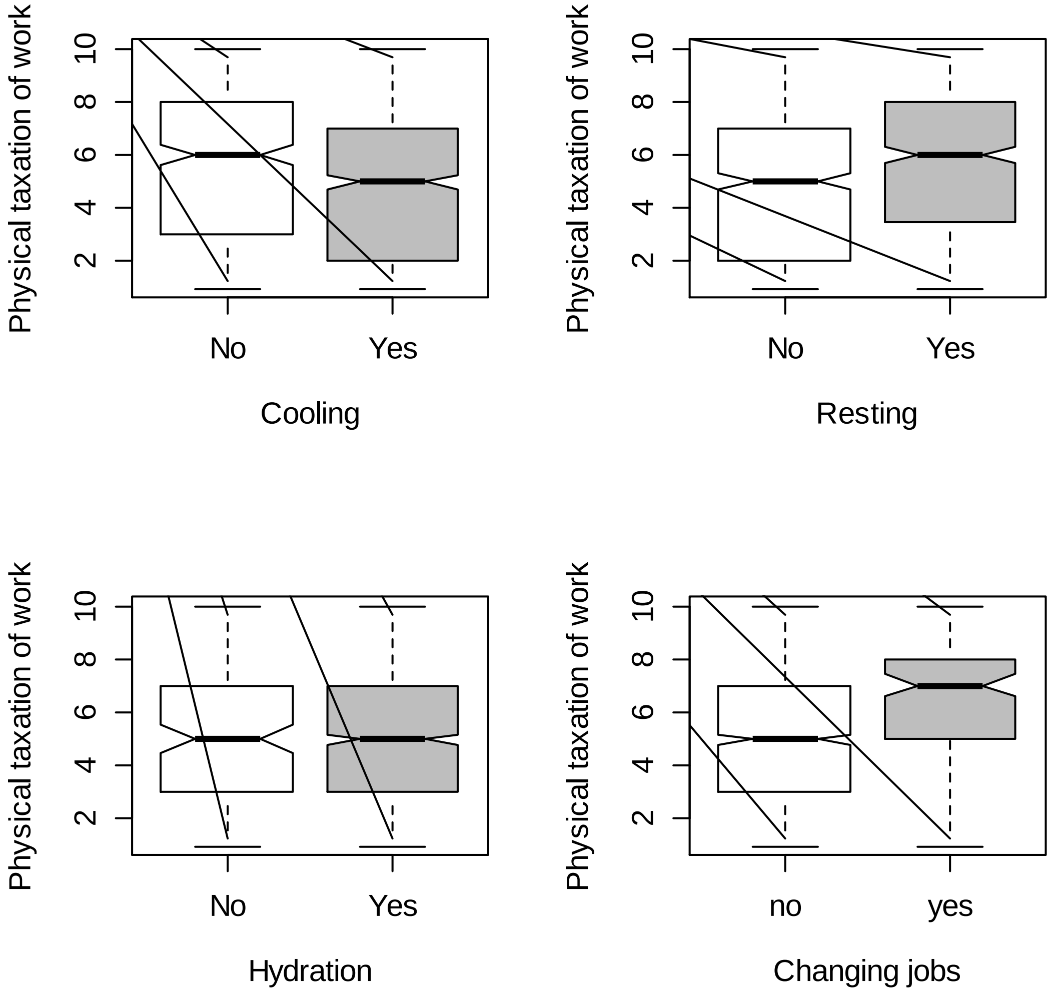

Figure S2. Heat relief measures taken by heat stressed respondents (N = 1719) at work by their levels of physical exertion at work.
